# The Role of Osteopontin as a Diagnostic and Prognostic Biomarker in Sepsis and Septic Shock

**DOI:** 10.3390/cells8020174

**Published:** 2019-02-18

**Authors:** Luigi Mario Castello, Marco Baldrighi, Luca Molinari, Livia Salmi, Vincenzo Cantaluppi, Rosanna Vaschetto, Greta Zunino, Marco Quaglia, Mattia Bellan, Francesco Gavelli, Paolo Navalesi, Gian Carlo Avanzi, Annalisa Chiocchetti

**Affiliations:** 1 Department of Translational Medicine, Università degli Studi del Piemonte Orientale, Via Solaroli 17, 28100 Novara (NO), Piedmont, Italy; marco.baldrighi590@gmail.com (M.B.); luca.molinari@med.uniupo.it (L.M.); livia.salmi@uniupo.it (L.S.); vincenzo.cantaluppi@med.uniupo.it (V.C.); rosanna.vaschetto@med.uniupo.it (R.V.); greta.fda@gmail.com (G.Z.); marco.quaglia@med.uniupo.it (M.Q.); bellanmattia@yahoo.it (M.B.); gavelli.francesco@gmail.com (F.G.); giancarlo.avanzi@med.uniupo.it (G.C.A.); 2 Emergency Department, “Maggiore della Carità” University Hospital, Corso Mazzini 18, 28100 Novara (NO), Piedmont, Italy; 3 CAAD, IRCAD, Department of Health Sciences, Università degli Studi del Piemonte Orientale, Corso Trieste 15, 28100 Novara (NO), Piedmont, Italy; annalisa.chiocchetti@med.uniupo.it; 4 Division of Nephrology and Transplantation, “Maggiore della Carità” University Hospital, Corso Mazzini 18, 28100 Novara (NO), Piedmont, Italy; 5 Division of Anesthesiology and Reanimation, “Maggiore della Carità” University Hospital, Corso Mazzini 18, 28100 Novara (NO), Piedmont, Italy; 6 Division of Internal Medicine, “Sant’Andrea” Hospital, Corso Abbiate 21, 13100 Vercelli (VC), Piedmont, Italy; 7 Division of Anesthesiology and Reanimation, Department of Medical and Surgical Sciences, Università degli Studi Magna Grecia, 88100 Catanzaro (CZ), Calabria, Italy; pnavalesi@gmail.com

**Keywords:** Osteopontin, biomarkers, sepsis, Emergency Department, diagnosis, risk stratification, prognosis

## Abstract

Sepsis is a life-threatening organ dysfunction caused by a dysregulated host-response to infections. Osteopontin (OPN) is an extracellular matrix protein involved in the inflammatory response. Our aim was to evaluate the diagnostic and prognostic performance in sepsis of a single OPN determination in the Emergency Department (ED). We conducted a single-centre prospective observational study in an Italian ED where we enrolled 102 consecutive patients presenting with suspected infection and qSOFA ≥ 2. OPN plasma concentration was found to be an independent predictor of sepsis (OR = 1.020, 95% CI 1.002–1.039, *p* = 0.031) and the diagnostic receiver operating characteristic (ROC) curve resulted in an area under the curve (AUC) of 0.878. OPN levels were positively correlated to plasma creatinine (r = 0.401 with *p* = 0.0001), but this relation was not explained by the development of acute kidney injury (AKI), since no difference was found in OPN concentration between AKI and non-AKI patients. The analysis of 30-days mortality showed no significant difference in OPN levels between alive and dead patients (*p* = 0.482). In conclusion, a single determination of OPN concentration helped to identify patients with sepsis in the ED, but it was not able to predict poor prognosis in our cohort of patients.

## 1. Introduction

Sepsis is a life-threatening organ dysfunction caused by a dysregulated host-response to infection, affecting about 13 million people every year worldwide, with mortality rates higher than 30% according to some reports [1].

Septic shock, on the other hand, identifies a subset of patients in which sepsis is associated with both persisting hypotension that requires vasopressors to maintain a mean arterial pressure ≥65 mmHg and a serum lactate level >2 mmol/L, despite adequate volume resuscitation [1]. The identification of organ dysfunction in patients with a suspected infection relies on an acute increase of two points in the Sequential Organ Failure Assessment (SOFA) score, which combines clinical and laboratory parameters suggestive of different organ impairments [2].

Nevertheless, sepsis and septic shock are time-dependent conditions in which a delay in antibiotic administration is associated with a significant increase in mortality rates, whereas prompt recognition and early treatment initiation are known to improve the patient’s outcome [3]. For these reasons, in the Emergency Department (ED) the SOFA score appears to have great limits of application, mostly related to the amount of time required to be completed, whilst the use of the quick-SOFA score (qSOFA) has been recommended for a rapid identification of septic patients [1].

In addition, several biomarkers have been proposed [4,5] to improve the accuracy of qSOFA and accelerate the diagnostic pathway as well as the prognostic stratification of septic patients in the ED. However, a benchmark is still lacking [6,7,8,9]. Among them, Osteopontin (OPN) seems to be a promising diagnostic tool, according to the results of previous studies on animal models and Intensive Care Unit (ICU) patients. OPN is an extracellular matrix protein involved in the inflammatory response: as an integrin-binding protein, it modulates leukocyte activation, migration and differentiation as well as cytokine secretion both in acute and chronic inflammation [10,11,12,13]. It has been shown that OPN circulating levels not only are elevated in sepsis [14], but they also progressively increase throughout Systemic Inflammatory Response Syndrome (SIRS), sepsis and septic shock [15] and they are associated with higher mortality rates both in animal models [16] and septic patients [15,17]. Furthermore, OPN has been demonstrated to be an independent predictor of both ICU and long-term mortality in critically ill patients (including septic patients); in particular, the prognostic power of OPN levels measured at day three from ICU admission has been found to be superior compared to the one of other routine biomarkers of inflammation, infection and organ failure such as C-reactive protein (CRP), international normalized ratio (INR) and procalcitonin (PCT) [17]. However, to date, little is known about the potential role of OPN as an early diagnostic biomarker of sepsis and septic shock, particularly in an ED setting.

The aim of this study was, thus, to evaluate if a single OPN determination at the first medical contact in the ED could be a useful tool for the emergency physician in the diagnostic workup and in the risk stratification of patients with sepsis.

## 2. Materials and Methods

### 2.1. Setting and Patients

This single-centre, prospective, observational, pilot study was performed in the ED of the “Maggiore della Carità” University Hospital in Novara (Italy) from October 2016 to March 2018. Patients presenting to the ED with suspected sepsis according to the Sepsis-3 criteria (suspected infection and qSOFA ≥2) [1] were consecutively enrolled. Exclusion criteria were pregnancy, age < 18 years and lack of a signed informed consent.

The study was conducted according to the guidelines of the local ethical committee in conformity to the principles of the Declaration of Helsinki and was prospectively registered at the Australian New Zealand Trials Registry (ACTRN12617000429358).

At the time of the ED visit, clinical data were recorded (including demographic characteristics, past medical history, vital signs, physical examination findings, laboratory results and imaging) and plasma and urine samples were collected, aliquoted and stored at −80 °C. Patients were then clinically re-evaluated after 24 and 48 h and at 7 days or at discharge. A telephone follow-up was performed at 30 days in order to assess mortality.

At the end of the study period, two expert emergency physicians (LMC and GCA) evaluated the clinical record of each patient in order to assess the final diagnosis according to the Sepsis-3 criteria. Therefore, patients were firstly divided in two groups depending on whether the suspected sepsis detected at ED admission was later confirmed (“sepsis”) or ruled out (“non-sepsis”). Then, septic patients were further divided in two groups (notably “sepsis” and “septic shock”) in order to perform additional analyses.

### 2.2. Experimental Analysis

OPN plasma levels were determined at the end of the study period in the stored samples collected at the time of enrollment. Circulating OPN was quantified using commercially available ELISA (enzyme-linked immunosorbent assay; R&D system codes DY1433 and DY007) according to the manufacturer’s protocol. The optimal sample dilution was set at 1:1000 and the measure unit was ng/mL.

OPN levels were determined in 10 healthy subjects with similar age and gender distributions as those in the study group in order to have a control group.

### 2.3. Statistical Analysis

Statistical analysis was performed with the MedCalc® software v12.5.0 (MedCalc software bvba, Ostend, Belgium). Continuous variables were analyzed through the Mann–Whitney U test and logistic regressions were then used to identify independent predictors of sepsis. The Chi-square test was used to analyze categorical variables. 

The area under the Receiver Operating Characteristic (ROC) curve for the ability of OPN to distinguish septic patients from the non-septic ones and its best cut-off were calculated. Subsequently, we performed a pairwise comparison of the ROC curve for OPN and for the variables that were significantly different between the sepsis and non-sepsis groups, using the DeLong method with the Bonferroni correction.

In order to exclude the presence of potential confounders, the relationships between OPN levels and a few relevant variables were analyzed in the overall population using the Mann–Whitney U test (for categorical variables) or the rank correlation (for continuous variables). A multiple regression analysis was then performed to identify independent predictors of OPN concentration.

Survival times were calculated starting from the time of patients’ enrollment: the log-rank test was used to identify groups with different survival probabilities, graphically presented in Kaplan–Meier plots; hazard ratios (HR) and 95% confidence intervals (CI) were also calculated. Eventually, a Cox proportional hazard model was built to analyze the weight of mortality predictors identified at univariate analysis.

Statistical significance was set at two-tailed *p* < 0.05.

## 3. Results

From October 2016 to March 2018, 102 consecutive patients with suspected sepsis were enrolled in this study. One of them retired his consent soon after enrollment; the final analysis therefore included 101 patients.

The main characteristics of the overall population are reported in Table 1.

In 9 out of 101 patients (8.9%) sepsis was eventually ruled out, with the alternative definitive diagnosis being either non-infectious conditions (66.7%) or infections without organ dysfunction (33.3%): specifically, these alternative diagnosis were acute heart failure (33.3%), pulmonary embolism (22.2%), uncomplicated urinary tract infection (22.2%), ischemic bowel disease (11.1%) and dehydration due to acute diarrhea without sepsis (11.1%). Among the remaining 92 subjects (91.1%), 68 were diagnosed with sepsis (73.9%) and 24 with septic shock (26.1%).

### 3.1. Diagnosis

The comparison between septic (including both sepsis and septic shock) and non-septic patients showed significant differences in terms of SOFA score (6 vs. 4, *p* = 0.021), heart rate (110 vs. 80 bpm, *p* = 0.029), peripheral oxygen saturation (90% vs. 96%, *p* = 0.009) and plasma lactate concentration (2.8 vs. 0.8 mmol/L, *p* < 0.001). Body mass index (BMI) and respiratory rate (RR) were different at a non-significant level. Plasma OPN levels were found to be significantly higher in patients with sepsis (225.2 vs. 91.3 ng/mL, *p* < 0.001) (see again Table 1 for all variables details). 

A logistic regression analysis including all the above-mentioned variables together with age showed that OPN was an independent predictor of sepsis (OR = 1.020, 95% CI 1.002 to 1.039 with *p* = 0.031). Moreover, higher BMIs reduced the probability that the patients actually had sepsis (OR = 0.656, 95% CI 0.455 to 0.947 with *p* = 0.024). None of the other included variables was an independent predictor of sepsis according to this model (Table 2).

**Figure 1:**
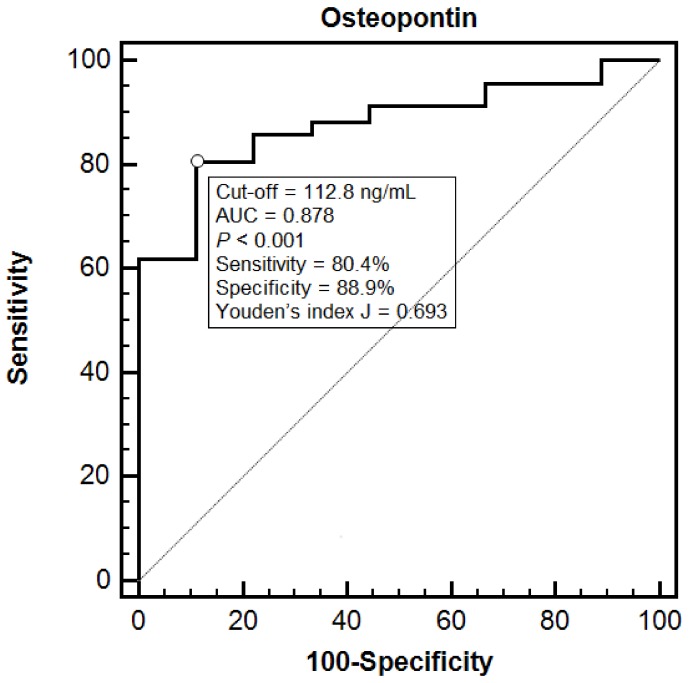
This figure represents the diagnostic performance of OPN in discriminating non-septic from septic patients. Receiving Operating Characteristic (ROC) curve analysis with area under the curve (AUC) was performed and reported in the figure.

The diagnostic performance of OPN was also evaluated through a ROC curve analysis (Figure 1). The area under the curve (AUC) was 0.878 (95% CI 0.798 to 0.935) and a cut-off of 112.8 ng/mL showed an 80.4% sensitivity and an 88.9% specificity in detecting septic patients (Youden’s index *J* = 0.693). The comparison between the ROC curve for OPN and those for heart rate, peripheral oxygen saturation, plasma lactate and SOFA score, showed no significant differences, being OPN AUC nearly identical to the plasma lactate one (0.877, 95% CI 0.796 to 0.934).

OPN levels were found to be slightly higher in patients with septic shock compared to those with sepsis, although this difference was not statistically significant (243.3 ng/mL vs. 211.6 ng/mL, *p* = 0.138). Circulating OPN was significantly lower in healthy controls compared to all the enrolled patients (35.2 vs. 204.6 ng/mL, *p* < 0.0001).

### 3.2. Independent Predictors of OPN Concentration

In order to identify the variables that could affect OPN circulating levels and act as potential confounders, the Mann–Whitney U test was used to compare OPN between males and females and between patients with or without certain comorbidities, while the rank correlation test was used to explore the association between OPN levels and a few relevant continuous variables (age, SOFA score, laboratory parameters). This analysis (reported in detail in Table 3) showed that, among the investigated variables, only plasma lactate (ρ = 0.370 with *p* = 0.0001), plasma creatinine (ρ = 0.262 with *p* = 0.008) and SOFA score (ρ = 0.243 with *p* = 0.014) were positively correlated to OPN.

However, when the three variables were included into a multiple regression analysis, only the increase in plasma creatinine turned out to be significantly associated to an increase in plasma OPN (r = 0.401 with *p* = 0.0001).

As reported in Table 1, no difference was found in creatinine levels between septic and non-septic patients (*p* = 0.311). Therefore, data obtained from septic patients were retrospectively reviewed in order to identify those who met the KDIGO (Kidney Disease Improving Global Outcome) criteria for acute kidney injury (AKI) [18]. Forty-seven out of 92 patients (51.1%) met the diagnostic criteria for AKI, but no significant difference emerged in OPN concentration between AKI and non-AKI patients (233.3 vs. 190.0 ng/mL, *p* = 0.119).

### 3.3. Mortality

Mortality rates at 30 days were evaluated dividing patients in three groups: non-sepsis, sepsis and septic shock. We observed a significant difference in mortality rates, which were progressively higher in non-septic, septic and septic shock patients (11.1% vs. 27.9% vs. 62.5%, respectively, *p* = 0.003) (Table 4).

**Figure 2:**
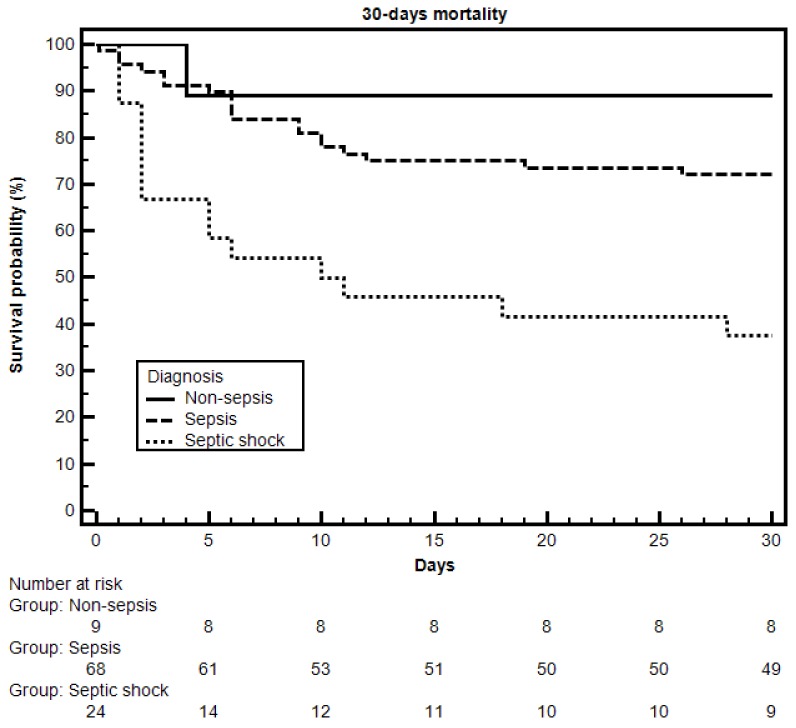
This figure shows the survival rates of the three diagnosis groups during the follow-up period of 30 days. Below the plot there is the “number at risk” table, indicating the number of patients alive in each group at every major timepoint.

Figure 2 reports the Kaplan–Meier curves that represent survival rates for the follow-up period of 30 days. Statistical significance was reached both when all three diagnosis were considered (*p* < 0.001) and when only sepsis and septic shock were compared (*p* < 0.001).

Considering only septic patients (including both sepsis and septic shock), GCS, CRP, arterial blood pH, plasma lactate, the PaO_2_/FiO_2_ ratio, qSOFA and SOFA turned out to be significantly different between alive and dead patients at 30 days; OPN concentration was not different between the two groups (231.3 ng/mL in dead patients vs. 216.8 ng/mL in alive patients, *p* = 0.482) (Table 5).

The Cox proportional hazard model built on the basis of this univariate analysis did not include OPN and is therefore provided in the Supplementary Material.

The same analysis was performed considering 7-days mortality, but once again OPN showed a poor prognostic performance and was not identified as an independent predictor of worse outcome (data available in the Supplementary Material).

## 4. Discussion

In this pilot study, performed on a cohort of 101 patients admitted to the ED over an 18-month period, we show that the baseline value of plasmatic OPN is a promising diagnostic biomarker for the early diagnosis of sepsis. Nevertheless, OPN did not act as a prognostic tool.

Although OPN levels have been previously studied in sepsis, our findings bring some elements of innovation. First of all, the role of OPN as a diagnostic biomarker had never been specifically investigated in the first approach to patients with suspected sepsis in an ED setting. Secondly, to our best knowledge this is the first study in which both the enrollment and the diagnostic classification of patients were performed according to the new definitions of sepsis and septic shock.

Among the 101 patients enrolled in the study, 92 of them showed a combination of infection and organ dysfunction, whereas nine had conditions that mimicked sepsis (including both non-infectious organ dysfunctions and uncomplicated infections). The comparison of these two groups revealed that OPN was significantly higher in septic patients.

Compared to the plasmatic levels reported in other studies, we observed lower OPN values [15,17]: on the one hand this could be explained by the different setting in which we performed the study (ED vs. ICU). On the other hand, the difference could be explained by the fact that we determined OPN levels at presentation, when the biohumoral modifications of sepsis are still developing.

Interestingly, OPN levels progressively increased throughout healthy controls, non-infectious conditions, sepsis and septic shock. This is coherent with the results of other studies [15] and proves that a pro-inflammatory state is present also in non-septic conditions and that OPN is involved in the pathogenesis of these inflammatory processes, since higher levels correspond to clinical syndromes with a more pronounced inflammatory dysregulation.

The correlation observed between OPN and creatinine plasma levels is in line with data from previous studies [19]. However, our hypothesis that this finding may be explained by a high rate of AKI in septic patients was not supported by the results: in fact, although OPN has been already described as a biomarker of AKI in critically ill patients [20], OPN levels did not differ significantly between AKI and non-AKI patients. Our data do not allow us to discriminate among a mere retentive effect caused by renal failure, an increase due to an active OPN secretion in an inflammatory setting or the coexistence of both these mechanisms which eventually lead to a vicious circle of accumulation and renal damage. However, further studies would be needed to deepen this issue and produce more conclusive results.

This biomarker resulted to be an independent diagnostic predictor of sepsis and showed a good diagnostic performance at the ROC curve analysis, where the highest sensitivity (88.9%) and specificity (80.4%) were seen when the cut-off was set at 112.8 ng/mL. However, when the ROC curve for OPN was compared to those for other potential markers of sepsis identified at univariate analysis (heart rate, peripheral oxygen saturation, plasma lactate and SOFA score), no significant difference was found. In particular, the AUC for OPN was very similar to the one for plasma lactate, a parameter widely and routinely used in the diagnostic workup and prognostic stratification of sepsis [21]. These data suggest that a single determination of plasma OPN in patients presenting with a suspected infection and a qSOFA score ≥ 2 can strengthen the suspicion of sepsis.

According to the design of this exploratory study, we did not enroll septic patients with qSOFA ≤ 1, which could have allowed us to evaluate the ability of OPN to increase the qSOFA sensitivity. However, the diagnostic value of OPN could be further tested in studies aimed at demonstrating whether it could increase the sensitivity of currently available clinical scores. In this context, by reinforcing the association between OPN and sepsis, our study opens new highly relevant scenarios.

In fact, OPN targeting might be considered a potential promising therapeutic strategy in septic patients. This is in line with previous reports, according to which OPN might play an important pathophysiologic role in the development of the systemic inflammation and the subsequent organ injury that characterizes sepsis. In a murine model of sepsis with acute lung injury (ALI), the neutralization of OPN with specific antibodies significantly reduced the circulating levels of pro-inflammatory cytokines, their mRNA expression in the lungs and even the levels of some biomarkers of organ injury [22].

Mortality rates at 30 days were not different between septic and non-septic patients. However, when the sepsis group was further divided in sepsis and septic shock, the comparison reached statistical significance as mortality rates were found to increase according to the severity of the disease. This latter observation was confirmed by the Kaplan–Meier curves reported in Figure 2.

The mortality rates observed in septic patients (including both sepsis and septic shock) were in line with those usually described in the literature. Nonetheless, mortality rates in septic shock alone were found to be higher than those reported in other studies [1]. This could be explained both by the characteristics of our population and by the study design itself. On the one hand, enrolled patients were mostly elderly affected by multiple comorbidities. On the other hand, the protocol of the study did not specifically exclude patients who had no indication to resuscitating procedures or intensive treatments because of their general condition, age and comorbidities, whereas many studies reported in the literature are designed to exclude “do not resuscitate” (DNR) patients (an international legal definition which has no equivalents in the Italian Law). This may have contributed to increase mortality rates in the septic shock group, since we retrospectively identified five patients (20.8% of all septic shock patients and 41.7% of the deaths observed within the first few days from admission) in which intensive treatments (such as admission to ICU, invasive monitoring, amine treatment and intensive resuscitation) were considered to be a form of futile medical care by the treating physicians.

The survival analysis showed that circulating OPN was not an independent predictor of mortality in our cohort of patients with sepsis. Actually, no significant difference was found in OPN levels between dead and alive patients at 30 days. This is not consistent with the data reported in the literature that, instead, attribute to OPN a good prognostic performance [17]. This may be explained by the timing of the determination of OPN levels in this study: subsequent measurements may have detected a difference that was not evident at the time of presentation. Therefore, the lack of further determination of OPN levels is another limitation of this study. In particular, having more information about the OPN time course might have allowed us to detect the peak of OPN concentration, known to be associated with increased mortality [15]. On the basis of our results we can only state that a single early OPN determination cannot be used as a prognostic tool. Eventually, another limitation of this pilot study is the small number of non-septic patients. However, due to the study design itself, the composition of the two groups remained unknown until the end of the study period, when the diagnostic workup of all the enrolled patients was completed. The inclusion criteria, as expected, allowed us to identify patients with sepsis with a good accuracy: the ratio between no sepsis and sepsis patients in our cohort was 1:10. In this regards, our results are in line with the findings of previously published studies [23].

## 5. Conclusions

A single determination of OPN at presentation can help to identify patients with sepsis in the ED, since its circulating levels are higher in sepsis compared to different acute conditions that can mimic sepsis. OPN concentration tends to increase according to the severity of the disease, being the highest in septic shock. Nevertheless, a single determination of OPN was not able to predict poor prognosis in this cohort of septic patients.

## Figures and Tables

**Table 1:** Main general, clinical and laboratory data of the study population. Data are presented for the whole population in the second column. The last three columns represent the data of patients with or without sepsis and their statistical comparison. Continuous variables are presented as medians and interquartile ranges; categorical variables are presented as frequencies (%).

	All Patients (N. 101)	Non-sepsis (N. 9)	Sepsis (N. 92)	*p*-Value
**General characteristics**				
**Age, years**	80 (73–88)	84 (64–90)	80 (73–88)	0.934
**Sex, M/F**	57 (56.4%)/44 (43.6%)	7 (77.8%)/2 (22.2%)	50 (54.3%)/42 (45.7%)	0.317
**BMI, kg/m^2^**	24.8 (22.0–27.5)	28.9 (24.6–31.2)	24.2 (22.0–27.1)	0.071
**Comorbidities**				
**Heart failure**	25 (24.8%)	3 (33.3%)	22 (24.0%)	0.826
**Previous stroke**	19 (18.8%)	1 (11.1%)	18 (19.6%)	0.863
**Dementia**	28 (27.7%)	2 (22.2%)	26 (28.3%)	0.997
**COPD**	17 (16.8%)	1 (11.1%)	16 (17.4%)	0.989
**Diabetes mellitus**	33 (32.7%)	1 (11.1%)	32 (34.8%)	0.283
**Neoplasia**	24 (23.8%)	1 (11.1%)	23 (25.0%)	0.600
**Arterial hypertension**	71 (70.3%)	7 (77.8%)	64 (69.6%)	0.895
**CKD**	31 (30.7%)	3 (33.3%)	28 (30.4%)	0.948
**Initiated antibiotic treatment ^‡^**	22 (21.8%)	1 (11.1%)	21 (22.8%)	0.674
**Clinical parameters**				
**HR, bpm**	107 (91–125)	80 (73–108)	110 (93–126)	**0.029** *
**MAP, mmHg**	73.3 (64.6–95.0)	76.3 (70.0–103.9)	73.3 (61.7–94.2)	0.270
**RR, breaths per minute**	30 (25–36)	26 (24–30)	30 (26–36)	0.056
**POS, %**	91 (85–95)	96 (94–98)	90 (85–95)	**0.009** *
**GCS**	13 (11–14)	13 (12–14)	13 (10–14)	0.316
**Body temperature, °C**	37.9 (37.3–38.8)	37.7 (36.0–38.5)	38.0 (37.3–38.8)	0.459
**Laboratory data**				
**WBCs, ×10^3^/mm^3^**	14.44 (9.46–20.40)	12.99 (10.00–16.56)	14.57 (9.26–21.78)	0.520
**Hb, g/dL**	12.3 (10.8–13.6)	12.1 (11.5–14.3)	12.4 (10.8–13.5)	0.962
**PLTs, ×10^3^/mm^3^**	217 (160–295)	206 (177–301)	218 (149–294)	0.757
**Glucose, mg/dL**	138 (104–191)	136 (122–148)	139 (103–217)	0.807
**Creatinine, mg/dL**	1.45 (0.94–2.12)	1.18 (0.88–1.66)	1.54 (0.94–2.15)	0.311
**Total bilirubin, mg/dL**	0.7 (0.5–1.3)	0.5 (0.5–1.0)	0.8 (0.5–1.4)	0.197
**CRP, mg/dL**	12.77 (3.12–19.40)	2.10 (1.40–14.67)	13.24 (3.40–19.57)	0.127
**Arterial pH**	7.44 (7.39–7.48)	7.44 (7.37–7.48)	7.44 (7.39–7.48)	0.826
**Plasma lactate, mmol/L**	2.5 (1.6–4.8)	0.8 (0.7–1.6)	2.8 (1.8–5.3)	**<0.001** *
**PaO_2_/FiO_2_, mmHg**	250.0 (210.2–321.6)	300.5 (225.2–358.0)	247.6 (210.0–313.4)	0.255
**OPN, ng/mL**	204.6 (112.5–376.8)	91.3 (63.9–105.4)	225.2 (138.2–387.8)	**<0.001** *
**Scores**				
**qSOFA, 2/3 ^†^**	69 (68.3%)/32 (31.7%)	7 (77.8%)/2 (22.2%)	62 (67.4%)/30 (32.6%)	0.792
**SOFA**	6 (4–7)	4 (2–5)	6 (4–7)	**0.021** *

* and **bold value** indicate statistical significance according to *p*-value < 0.05. ^‡^ patients were included in this group if antibiotic treatment had been already initiated before ED admission. ^†^ patients were divided according to the qSOFA score in two groups (qSOFA = 2 vs. qSOFA = 3). **Abbreviations list**: BMI: body mass index; COPD: chronic obstructive pulmonary disease; CKD: chronic kidney disease; HR: heart rate; MAP: mean arterial pressure; RR: respiratory rate; POS: peripheral oxygen saturation; GCS: Glasgow Coma Scale; WBCs: white blood cells, Hb: haemoglobin; PLTs: platelets; CRP: C-reactive protein; PaO_2_/FiO_2_: ratio between partial pressure of oxygen and fractional inspired oxygen; OPN: plasma Osteopontin concentration; SOFA: Sepsis-related Organ Failure Assessment; qSOFA: quick SOFA.

**Table 2:** Logistic regression model of the predictors of sepsis. The Table shows the OR resulted from multivariate analysis. Variables were selected if their *p*-values at univariate analysis were < 0.10 (see Table 1).

	OR	95% CI	*p*-Value
**Age**	0.976	0.876–1.087	0.656
**BMI**	0.656	0.455–0.947	**0.024** *
**HR**	1.003	0.937–1.074	0.934
**RR**	1.187	0.896–1.574	0.233
**POS**	1.008	0.808–1.256	0.946
**Plasma lactate**	4.546	0.500–41.303	0.179
**OPN**	1.020	1.002–1.039	**0.031** *
**SOFA**	1.799	0.840–3.849	0.131

* and **bold value** indicate statistical significance according to *p*-value < 0.05. **Abbreviations list**: OR: odds ratio; CI: confidence interval; BMI: body mass index; HR: heart rate; RR: respiratory rate; POS: peripheral oxygen saturation; OPN: plasma Osteopontin concentration; SOFA: Sepsis-related Organ Failure Assessment score.

**Table 3:** Univariate analysis of potential predictors of OPN levels. Patients were divided into two groups according to sex and to the presence or absence of certain comorbidities and the Mann–Whitney U test was used to compare OPN between them (the first column reports median and interquartile range of OPN concentration in the groups). The rank correlation was used to investigate the relationship between OPN levels and a few relevant continuous variables (the second column reports the Spearman’s ρ, while the third reports the 95% CI). The forth column reports the *p*-value for each test.

		OPN, ng/mL	ρ	95% CI	*p*-Value
**Categorical Variables**					
**Sex**	**M**	191.3 (105.7–353.7)			0.511
	**F**	226.0 (123.0–387.8)			
**Heart failure**	**Y**	190.0 (107.3–237.8)			0.325
	**N**	225.2 (117.3–387.8)			
**Previous stroke**	**Y**	226.1 (115.0–355.6)			0.768
	**N**	193.6 (112.8–378.8)			
**Dementia**	**Y**	228.0 (149.0–354.6)			0.750
	**N**	193.1 (106.1–386.7)			
**COPD**	**Y**	215.4 (107.5–390.5)			0.806
	**N**	193.6 (117.1–377.5)			
**Diabetes mellitus**	**Y**	204.6 (125.6–380.5)			0.789
	**N**	200.2 (112.2–354.6)			
**Neoplasia**	**Y**	225.3 (165.3–411.0)			0.128
	**N**	190.0 (107.7–350.8)			
**Arterial hypertension**	**Y**	206.3 (122.8–384.0)			0.542
	**N**	192.0 (99.1–345.9)			
**CKD**	**Y**	189.9 (108.8–344.1)			0.581
	**N**	207.0 (122.6–385.7)			
**Continuous variables**					
**SOFA**			0.243	0.050–0.418	**0.014** *
**Age, years**			0.112	−0.085–0.301	0.264
**BMI, kg/m^2^**			−0.038	−0.233–0.161	0.711
**Creatinine, mg/dL**			0.262	0.070–0.435	**0.008** *
**PaO_2_/FiO_2_, mmHg**			−0.139	−0.326–0.058	0.165
**WBCs, ×10^3^/mm^3^**			0.070	−0.128–0.262	0.489
**PLTs, ×10^3^/mm^3^**			0.003	−0.192–0.198	0.975
**CRP, mg/dL**			0.161	−0.036–0.345	0.108
**Arterial pH**			−0.114	−0.303–0.083	0.257
**Plasma lactate, mmol/L**			0.370	0.188–0.527	**0.0001** *

* and **bold value** indicate statistical significance according to *p*-value < 0.05. **Abbreviations list**: OPN: plasma Osteopontin concentration; COPD: chronic obstructive pulmonary disease; CKD: chronic kidney disease; SOFA: Sepsis-related Organ Failure Assessment; BMI: body mass index; PaO_2_/FiO_2_: ratio between partial pressure of oxygen and fractional inspired oxygen; WBCs: white blood cells; PLTs: platelets; CRP: C-reactive protein.

**Table 4:** This table represents mortality rates at 30 days according to the different diagnosis groups.

	**Non-sepsis (N. 9)**	**Sepsis (N. 92)**		***p*-Value**
**30-days mortality**	1/9 (11.1%)	34/92 (37.0%)		0.235
	**Non-sepsis (N. 9)**	**Sepsis (N. 68)**	**Septic shock (N. 24)**	***p*-Value**
**30-days mortality**	1/9 (11.1%)	19/68 (27.9%)	15/24 (62.5%)	**0.003** *

* and **bold value** indicate statistical significance according to *p*-value < 0.05 for Chi-square test.

**Table 5:** Main general, clinical and laboratory data of the 92 patients with sepsis divided according to being alive or dead at 30 days. Continuous variables are presented as medians and interquartile range; categorical variables are presented as frequencies (%).

	Alive at 30 days (N. 58)	Dead at 30 days (N. 34)	*p*-Value
**General characteristics**			
**Age, years**	80 (71–86)	84 (75–89)	0.175
**Sex, M/F**	33 (56.8%)/25 (43.2%)	17 (50.0%)/17 (50.0%)	0.671
**BMI, kg/m^2^**	25.0 (22.2–27.3)	24.1 (21.3–26.1)	0.283
**Comorbidities**			
**Heart failure**	14 (24.1%)	8 (23.5%)	0.852
**Previous stroke**	8 (13.8%)	10 (29.4%)	0.121
**Dementia**	17 (29.3%)	9 (26.5%)	0.958
**COPD**	11 (19.0%)	5 (14.7%)	0.814
**Diabetes mellitus**	20 (34.5%)	12 (35.3%)	0.882
**Neoplasia**	15 (25.8%)	8 (23.5%)	0.999
**Arterial hypertension**	42 (72.4%)	22 (64.7%)	0.589
**CKD**	17 (29.3%)	11 (32.4%)	0.880
**Initiated antibiotic treatment ^‡^**	14 (24.1%)	7 (20.6%)	0.824
**Clinical parameters**			
**HR, bpm**	107 (92–125)	113 (94–127)	0.710
**MAP, mmHg**	73.3 (61.7–95.0)	73.3 (61.7–93.3)	0.900
**RR, breaths per minute**	28 (25–35)	32 (26–40)	0.080
**POS, %**	91 (86–95)	89 (81–94)	0.122
**GCS**	13 (11–14)	12 (9–13)	**0.010** *
**Body temperature, °C**	38.0 (37.3–38.9)	37.8 (37.2–38.7)	0.201
**Laboratory data**			
**WBCs, ×10^3^/mm^3^**	13.87 (8.30–20.86)	15.09 (9.80–24.16)	0.656
**Hb, g/dL**	12.3 (10.9–13.5)	12.6 (10.5–13.5)	0.815
**PLTs, ×10^3^/mm^3^**	218 (163–294)	216 (128–290)	0.680
**Glucose, mg/dL**	142 (108–190)	131 (90–224)	0.332
**Creatinine, mg/dL**	1.39 (0.94–2.09)	1.66 (1.08–2.39)	0.280
**Total bilirubin, mg/dL**	0.7 (0.6–1.4)	0.8 (0.3–1.4)	0.491
**CRP, mg/dL**	10.36 (2.92–17.20)	15.72 (6.21–19.94)	**0.046** *
**Arterial pH**	7.44 (7.42–7.49)	7.40 (7.36–7.46)	**0.012** *
**Plasma lactate, mmol/L**	2.4 (1.6–4.0)	3.2 (2.1–6.8)	**0.029** *
**PaO_2_/FiO_2_, mmHg**	260.0 (230.0–336.7)	234.5 (182.2–259.5)	**0.023** *
**OPN, ng/mL**	216.8 (144.0–356.6)	231.3 (123.4–526.4)	0.482
**Scores**			
**qSOFA, 2/3** **^†^**	44 (75.9%)/14 (24.1%)	18 (52.9%)/16 (47.1%)	**0.042** *
**SOFA**	5 (4–7)	7 (5–8)	**0.023** *

* and **bold value** indicate statistical significance according to *p*-value < 0.05. ^‡^ patients were included in this group if antibiotic treatment had been already initiated before ED admission. ^†^ patients were divided according to the qSOFA score in two groups (qSOFA = 2 vs. qSOFA = 3). **Abbreviations list**. BMI: body mass index; COPD: chronic obstructive pulmonary disease; CKD: chronic kidney disease; HR: heart rate; MAP: mean arterial pressure; RR: respiratory rate; POS: peripheral oxygen saturation; GCS: Glasgow Coma Scale; WBCs: white blood cells, Hb: haemoglobin; PLTs platelets; CRP: C-reactive protein; PaO_2_/FiO_2_: ratio between partial pressure of oxygen and fractional inspired oxygen; OPN: plasma Osteopontin concentration; SOFA: Sepsis-related Organ Failure Assessment; qSOFA: quick SOFA.

## Supplementary Materials

The following are available online at https://www.mdpi.com/2073-4409/8/2/174/s1. Supplementary Materials provide further data about the analysis of the prognostic role of OPN in sepsis.

## Abbreviations

OPN: Osteopontin; ED: Emergency Department; ROC: receiving operating characteristic; AUC: area under the curve; SOFA: Sequential Organ Failure Assessment; ICU: Intensive Care Unit; qSOFA: quick SOFA; SIRS: Systemic Inflammatory Response Syndrome; CRP: C-reactive protein; INR: international normalized ratio; PCT: procalcitonin; ELISA: enzyme-linked immunosorbent assay; HR: hazard ratio; CI: confidence interval; BMI: body mass index; COPD: chronic obstructive pulmonary disease; CKD: chronic kidney disease; HR: heart rate; MAP: mean arterial pressure; RR: respiratory rate; POS: peripheral oxygen saturation; GCS: Glasgow Coma Scale; WBCs: white blood cells, Hb: haemoglobin; PLTs: platelets; PaO_2_/FiO_2_: ratio between partial pressure of oxygen and fractional inspired oxygen; OR: odds ratio; KDIGO: Kidney Disease Improving Global Outcome; AKI: acute kidney injury; ALI: acute lung injury.
