# Supplementary material for: The Role of Osteopontin as a Diagnostic and Prognostic Biomarker in Sepsis and Septic Shock
Source: Cells. 2019 Feb 18;8(2):174. doi: 10.3390/cells8020174 (PMC6407102; doi:10.3390/cells8020174)
Supplement: Supplementary file 1 [file cells-08-00174-s001.pdf]

Independent predictors of 30-days mortality

On the basis of the univariate analysis reported in **Table 3** in the Manuscript, a Cox proportional hazard regression model identified as independent predictors of 30-days mortality the GCS score (HR = 0.879, *P* = 0.043), CRP levels (HR = 1.061, *P* = 0.003), arterial blood pH (HR = 0.008, *P* = 0.026), the plasma lactate concentration (HR = 1.172, *P* = 0.017) and the PaO_2/FiO_2 ratio (HR = 0.995, *P*= 0.038) (**Table S1**).

Table S1. Cox proportional hazard regression model of the factors associated with 30-days mortality. The Table shows the HR resulted from multivariate analysis. Variables were selected if their *P*-values at univariate analysis of 30-days mortality was < 0.10.

Table S1	HR	95% CI	*P*-value
**Age**	1.035	0.991 - 1.080	0.122
**Sex #**	1.426	0.619 - 3.281	0.407
**RR**	1.007	0.963 - 1.054	0.761
**GCS**	0.879	0.776 - 0.995	**0.043***
**CRP**	1.061	1.021 - 1.103	**0.003***
**Arterial pH**	0.008	0.001 - 0.541	**0.026***
**Plasma lactate**	1.172	1.030 - 1.335	**0.017***
**PaO_2/FiO_2**	0.995	0.991 - 0.999	**0.038***
**qSOFA = 3 #**	0.997	0.436 - 2.276	0.993
**SOFA**	1.017	0.842 - 1.227	0.866

* and **bold value** indicate statistical significance according to *P*-value < 0.05.
# categorical variables.
Abbreviations list: HR: hazard ratio; CI: confidence interval; RR: respiratory rate; GCS: Glasgow Coma Scale; CRP: C-reactive protein; PaO_2/FiO_2: ratio between partial pressure of oxygen and fractional inspired oxygen; SOFA: Sepsis-related Organ Failure Assessment score; qSOFA= 3: quick SOFA score = 3 points.

Analysis of 7-days mortality

Seven-days mortality progressively increased in non-septic patients, sepsis and septic shock (respectively, 11.1% vs. 16.2% vs. 45.8% with *P* = 0.008; **Table S2**).

Table S2. This table represents the different mortality rates at 7 days according to different diagnosis groups.

Table S2	Non-sepsis (N. 9)	Sepsis (N. 92)		*P*-value
**7-days mortality**	1/9 (11.1%)	22/92 (23.9%)		0.647
	**Non-sepsis (N. 9)**	**Sepsis (N. 68)**	**Septic shock (N. 24)**	***P*-value**
**7-days mortality**	1/9 (11.1%)	11/68 (16.2%)	11/24 (45.8%)	**0.008***

* and **bold value** indicate statistical significance according to *P*-value < 0.05 for Chi-square test.

Considering only sepsis patients (including both sepsis as such and septic shock), RR, blood glucose, arterial pH, plasma lactate concentration and the PaO_2/FiO_2 ratio turned out to be significantly different between alive and dead patients at 7 days; OPN concentration was higher in patients who died compared to those who survived without reaching statistical significance (268.2 ng/mL vs. 207.0 ng/mL, *P* = 0.082) (**Table S3**).

Table S3. Main general, clinical and laboratory data of the 92 sepsis patients divided according to being alive or dead at 7 days. Continuous variables are presented as medians and interquartile range; categorical variables are presented as frequencies (%).

Table S3	Alive at 7 days (N. 70)	Dead at 7 days (N. 22)	*P*-value
**General characteristics**			

**Age, years**	80 (71 - 87)	86 (75 - 89)	0.254
**Sex, M / F**	40 (57.1%) / 30 (42.9%)	10 (45.5%) / 12 (55.5%)	0.475
**BMI, kg/m^2**	24.6 (22.1 - 27.2)	24.1 (21.3 - 25.9)	0.508
**Anamnestic data**			
**Heart failure**	16 (22.9%)	6 (27.3%)	0.891
**Previous stroke**	13 (18.6%)	5 (22.7%)	0.904
**Dementia**	23 (32.9%)	3 (13.6%)	0.140
**COPD**	13(18.6%)	3 (13.6%)	0.834
**Diabetes mellitus**	25 (35.7%)	7 (31.8%)	0.938
**Neoplasia**	18 (25.7%)	5 (22.7%)	1.000
**Arterial hypertension**	48 (68.6%)	16 (72.7%)	0.917
**CKD**	19 (27.1%)	9 (40.9%)	0.329
**Initiated antibiotic treatment ‡**	18 (25.7%)	3(13.6%)	0.343
**Clinical parameters**			
**HR, bpm**	107 (92- 125)	114 (96 – 126)	0.481
**MAP, mmHg**	73 (62 - 95)	74 (62 - 90)	0.704
**RR, bpm**	28 (25 - 35)	33 (26 – 40)	**0.030***
**POS, %**	90 (85 - 95)	90 (86 - 94)	0.801
**GCS**	13 (11 - 14)	13 (10 - 14)	0.417
**Body temperature, °C**	38 (37.3 - 38.8)	37.8 (37.2 - 38.1)	0.248
**Laboratory data**			
**WBCs, $x10^3/mm^3$**	14.26 (9.21 - 22.70)	14.97 (9.27 - 20.26)	0.898
**Hb, g/dL**	12.4 (10.9 - 13.8)	12.4 (10.2 - 13.4)	0.413
**PLTs, $x10^3/mm^3$**	220 (165 - 296)	200 (126 - 290)	0.268
**Glucose, mg/dL**	143 (116 - 218)	118 (77 - 172)	**0.043***
**Creatinine, mg/dL**	1.49 (0.94 - 2.13)	1.58 (1.08 - 2.32)	0.564
**Total bilirubin, mg/dL**	0.7 (0.5 - 1.3)	0.9 (0.4 - 1.7)	0.945
**CRP, mg/dL**	11.0 (3.1 - 17.2)	16.1 (4.8 - 20.8)	0.090
**Arterial pH**	7.44 (7.42 - 7.49)	7.38 (7.33 - 7.45)	**0.001***
**Plasma lactate, mmol/L**	2.6 (1.6 - 3.9)	4.9 (2.1 - 9.0)	**0.013***
**PaO_2/FiO_2**	255.5 (224.3 - 320.5)	228.8 (144.8 - 251.9)	**0.033***
**OPN, ng/mL**	207.0 (130.6 - 356.6)	268.2 (162.6 - 528.4)	0.082
**Scores**			
**qSOFA, score 2 / 3 †**	50 / 20	12 / 10	0.225
**SOFA Score**	6 (4 - 7)	7 (5 - 8)	0.093

* and **bold value** indicate statistical significance according to P-value < 0.05.

‡ patients were included in this group if antibiotic treatment had been already initiated before ED admission.

† patients were divided according to the qSOFA score in two groups (qSOFA = 2 vs. qSOFA = 3).

Abbreviations list. BMI: body mass index; COPD: chronic obstructive pulmonary disease; CKD: chronic kidney disease; HR: heart rate; MAP: mean arterial pressure; RR: respiratory rate; POS: pulse oximetry saturation; GCS: Glasgow Coma Scale; WBC: white blood cells, Hb: haemoglobin; PLT: platelets; CRP: C-reactive protein; PaO_2/FiO_2 : ratio between partial pressure of oxygen and fractional inspired oxygen; OPN: plasma Osteopontin concentration; SOFA: Sepsis-related Organ Failure Assessment; qSOFA: quick SOFA.

A multivariate analysis including the above-mentioned variables together with age, sex, CRP levels and SOFA score identified as independent predictors of 7-days mortality in sepsis patients the plasma lactate concentration (HR = 1.215, *P* = 0.019), the PaO_2/FiO_2 ratio (HR = 0.990, *P* = 0.004) and CRP levels (HR = 1.086, *P* = 0.001); OPN showed a poor independent prognostic performance (HR 1.000, *P* = 0.939) (**Table S4**).

Table S4. Cox proportional hazard regression model of factors associated with 7-days mortality. The Table shows the Hazard Ratios resulted from multivariate analysis. Variables were selected if their p values at univariate analysis of mortality at 7 and at 30 days was with P-value <0.10.

Table S4	HR	95% CI	*P*-value

**Age**	1.045	0.989 - 1.105	0.118
**Sex #**	2.817	0.951 - 8.342	0.063
**RR**	0.999	0.939 - 1.063	0.978
**Glucose**	0.993	0.985 - 1.001	0.094
**CRP**	1.086	1.034 - 1.141	**0.001***
**Arterial pH**	0.015	0.001 - 3.112	0.125
**Plasma lactate**	1.215	1.033 - 1.429	**0.019***
**PaO_2/FiO_2**	0.990	0.983 - 0.997	**0.004***
**SOFA**	1.023	0.828 - 1.267	0.830
**OPN**	1.000	0.999 - 1.001	0.935

* and **bold** indicate statistical significance according to *P*-value < 0.05.

# categorical variables.

Abbreviations list: HR: hazard ratio; CI: confidence interval; RR: respiratory rate; CRP: C-reactive protein; PaO_2/FiO_2: ratio between partial pressure of oxygen and fractional inspired oxygen; SOFA: Sepsis-related Organ Failure Assessment score; OPN: plasma Osteopontin concentration).
